# A Cost Analysis of School-Based Lifestyle Interventions

**DOI:** 10.1007/s11121-018-0918-1

**Published:** 2018-05-31

**Authors:** Marije Oosterhoff, Hans Bosma, Onno C.P. van Schayck, Manuela A. Joore

**Affiliations:** 1 Department of Clinical Epidemiology and Medical Technology Assessment (KEMTA), Maastricht University Medical Centre MUMC+/Care and Public Health Research Institute (CAPHRI), Maastricht University, Maastricht, The Netherlands; 2 Department of Social Medicine, Care and Public Health Research Institute (CAPHRI), Faculty of Health, Medicine and Life Sciences, Maastricht University, P.O. Box 5800, 6202 AZ Maastricht, The Netherlands; 3 Department of Family Medicine, Care and Public Health Research Institute (CAPHRI), Faculty of Health, Medicine and Life Sciences, Maastricht University, Maastricht, The Netherlands

**Keywords:** Costs and cost analysis, School health services/economics, Schools, Child

## Abstract

A uniform approach for costing school-based lifestyle interventions is currently lacking. The objective of this study was to develop a template for costing primary school-based lifestyle interventions and apply this to the costing of the “Healthy Primary School of the Future” (HPSF) and the “Physical Activity School” (PAS), which aim to improve physical activity and dietary behaviors. Cost-effectiveness studies were reviewed to identify the cost items. Societal costs were reflected by summing up the education, household and leisure, labor and social security, and health perspectives. Cost inputs for HPSF and PAS were obtained for the first year after implementation. In a scenario analysis, the costs were explored for a hypothetical steady state. From a societal perspective, the per child costs were €2.7/$3.3 (HPSF) and €− 0.3/$− 0.4 (PAS) per day during the first year after implementation, and €1.0/$1.2 and €− 1.3/$− 1.6 in a steady state, respectively (2016 prices). The highest costs were incurred by the education perspective (first year: €8.7/$10.6 (HPSF) and €4.0/$4.9 (PAS); steady state: €6.1/$7.4 (HPSF) and €2.1/$2.6 (PAS)), whereas most of the cost offsets were received by the household and leisure perspective (first year: €− 6.0/$− 7.3 (HPSF) and €− 4.4/$− 5.4 (PAS); steady state: €− 5.0/$− 6.1 (HPSF) and €− 3.4/$− 4.1 (PAS)). The template proved helpful for costing HPSF and PAS from various stakeholder perspectives. The costs for the education sector were fully (PAS) and almost fully (HPSF) compensated by the savings within the household sector. Whether the additional costs of HPSF over PAS represent value for money will depend on their relative effectiveness.

**Electronic supplementary material:**

The online version of this article (10.1007/s11121-018-0918-1) contains supplementary material, which is available to authorized users.

## Introduction

It is increasingly being recognized that schools have a key role in the promotion of children’s health and wellbeing (Hayman 2016; Tang et al. 2009). A number of studies found that recent primary school-based lifestyle interventions are effective in normalizing children’s body mass index (BMI) (Mei et al. 2016; Sobol-Goldberg, Rabinowitz, and Gross 2013). Much less is known about the costs of these interventions (John, Wenig, and Wolfenstetter 2010). This study focuses on performing systematic and standardized cost calculations on school-based lifestyle interventions.

A wide range of cost estimates have been published, ranging from €4 to €865 per child per year (adjusted for purchase power parity) (see for example: (Barrett et al. 2015; Cradock et al. 2017; McAuley et al. 2010; te Velde et al. 2011; Waters et al. 2017), which is likely due to the high variation in intervention components, setup of interventions, and the heterogeneity in costing approaches. The heterogeneity in costing approaches is, amongst others, reflected by the various methods for identifying and selecting cost items. While the recurring resource use of intervention delivery is generally included in cost calculations, a minority of studies included development and evaluation costs, and consensus on this aspect seems to be lacking (Meng et al. 2013; Moodie et al. 2013; Wang, Li, Siahpush, Chen, and Huberty 2017). In addition to the variability in costing methods, most studies provided little information on how resource consumption was identified, measured, and valued. For example, most studies listed the included cost categories but did not justify why cost categories were excluded (e.g., not applicable to the intervention, not in alignment with the perspective of the costing study). The lack of standardization and transparency makes it difficult to ascertain whether cost estimates are valid and reliable. Standardized and transparent cost calculations are of utmost importance to be able to perform cost comparisons between interventions, and to examine which interventions yield the best value in return for the required investments. The methodological quality of cost estimations is also key for implementation as underestimating the intervention costs will lead to implementation, but delivery will not be sustained when the costs are found to be higher than initially estimated. In contrast, overestimating the intervention costs may withhold children from receiving the benefits of effective interventions.

Decision-makers in public health are interested in the social value of school interventions given the fact that most recent school-based lifestyle interventions are aimed at involving multiple stakeholders (e.g., schools, local government, sport clubs, caterers, child care organizations, health staff), and may affect outcomes in others than the child (Middleton, Evans, Keegan, Bishop, and Evans 2014; Warwick et al. 2005). Additionally, interventions may not only impact on health outcomes, but may also improve wellbeing by creating positive school environments, and improve educational outcomes as a result of the associations with children’s health status (Langford et al. 2014). Most economic evaluations have examined the social value of school-based lifestyle interventions by adopting a societal perspective for the calculation of costs and outcomes. In a societal perspective, all the costs and outcomes that change as a result of the intervention are considered, regardless of who incurs them or in which sector they fall. A previous study has identified that the costs and outcomes of public health interventions may fall into the healthcare, education, labor and social security, household and leisure, or safety and justice sector (Drost, Paulus, Ruwaard, and Evers 2014). To effectively inform decision-makers, it is important to perform cost calculations from a societal perspective and take account of the various sectors that are involved or affected by the delivery of school-based lifestyle interventions.

To determine the support to an intervention, stakeholders require information on the costs they will incur. So far, few studies distinguished different stakeholder perspectives, but doing so may be helpful to interpret whether the implementation of an intervention is economically feasible or whether a cost redistribution among stakeholder groups would facilitate implementation (Moodie, Carter, Swinburn, and Haby 2010; Moodie, Haby, Galvin, Swinburn, and Carter 2009; Moodie, Haby, Swinburn, and Carter 2011; Moodie et al. 2013).

The objective of this study was to develop a template for the costing of primary school-based lifestyle interventions. The template should enable to perform cost calculations from various stakeholder perspectives and examine the impact of different scenarios, assumptions, or intervention adaptations. To illustrate the applicability of this framework, we calculated the costs of the “Healthy Primary School of the Future” (HPSF) and the “Physical Activity School” (PAS).

## Methods

Firstly, we developed a template for the cost calculation of school-based lifestyle interventions. The template was then used for the cost calculation of HPSF and PAS by selecting the cost items that applied to both interventions and by measuring and valuing the selected cost items.

### Template for Cost Calculation

The cost items of primary school-based lifestyle interventions were identified from published cost-effectiveness studies on these type of programs (Kersley and Knuutila 2011; Oosterhoff et al. 2017). Cost items reflected the incremental resource consumption relative to the regular education program. We focused on the recurrent activities for implementation, delivery, and evaluation. The one-time investments for intervention development and scientific evaluation fell beyond the scope of this study. It was aimed to include all cost items that reflected the social opportunity costs of an intervention. Social opportunity cost takes account of the actual payments and the economic costs. The economic costs represent the value of all resources that are not billed, but which are used for the intervention and are thus no longer available for other purposes (e.g., time investments of volunteers). Cost items could reflect a positive cost (cost increase compared to the regular education program) or a negative cost (cost saving compared to the regular education program). Cost items with a negative cost should be directly related to intervention delivery and *not* to the outcomes of school-based lifestyle interventions (e.g., provision of lunches at school is a cost offset for the household). We refer to this type of cost as a delivery-related offset. In this study, we made a distinction between a societal perspective and various smaller perspectives, which contained of the healthcare, education, labor and social security, and the household and leisure perspective (Drost, Paulus, Ruwaard, and Evers 2014). Cost items were also classified on the basis of personnel and material costs.

### The HPSF and PAS Interventions

The HPSF and PAS interventions are being evaluated in a quasi-experimental study located in the southern region of the Netherlands. The interventions are delivered from October 2015 onwards to children between 4 and 12 years of age (Willeboordse et al. 2016). The HPSF (two schools) and PAS schools (two schools) both target at physical activity and involve a longer lunch break. At PAS, the lunch break is extended with approximately half an hour, while at HPSF, the lunch break is prolonged with about 1 hour. Therefore, children attend school to approximately 15:30/15:45 instead of 15:00. At HPSF, some of the lunch breaks involve an educational component to meet the education hour requirements. With regard to physical activity, additional sports, play, and creative activities are provided for at least 4 days a week during the lunch break. Activities are being set up by cross-discipline coordinators. Activities are guided by pedagogical staff from childcare partners with the assistance of volunteers. At HPSF, children are also provided with a healthy lunch and a morning snack. Lunches are prepared at schools by catering personnel and beneficiaries of unemployment benefits as part of their reintegration to the labor market. The lunches consist of a variety of food products from which children can choose. Lunch is provided in the classroom or in a central location in the school. The lunch sessions are supervised by pedagogical staff and volunteers. Schools were able to implement additional activities based on a list of relevant and evidence-based additional activities (Willeboordse et al. 2016). In this study, we only focused on the compulsory changes and did not examine the cost of additional activities. Control schools maintained the regular school curriculum. Further details have been published elsewhere (Willeboordse et al. 2016).

### Cost Analysis of HPSF and PAS

We examined the incremental costs, which is defined as the additional costs of HPSF and PAS in comparison to the regular school curriculum. The annual social opportunity costs of HPSF and PAS were calculated for the first year after their implementation. Key stakeholders were consulted by means of semi-structured interviews to examine which of the cost items from the template applied to HPSF and PAS, and to complement the template. A calculation sheet was created to incorporate the cost items, volume, and unit prices. The inputs for volume and unit prices were obtained retrospectively from primary data sources (budget information, accounting data, stakeholder interviews, curriculum information). Because the activities at HPSF and PAS were all provided in addition to the regular school curriculum, the resource use at HPSF and PAS was examined, and the resource use in control schools was set to zero. Volumes were based on the average consumption at HPSF and PAS. For the unit prices, we used both primary data sources and secondary data sources, with the latter mainly being used to obtain values for resources that were not directly billed (e.g., time investments of volunteers). Because the school hours were extended with approximately half an hour for 4 days a week, it was assumed that the primary caregiver of the child (usually a parent) would spend less time on providing after-school care (reduction in caregiver time). We included the value of this time offset, because the primary caregiver could use this time offset for work or leisure activities. For the calculations, we assumed that the average family size amounted to two children per household. Beneficiaries of unemployment benefits were employed to help at HPSF with preparing school the lunches. This resulted in a saving for the household sector due to the difference between salary and unemployment benefits for beneficiaries. In addition, employing beneficiaries resulted in a cost offset for the social security sector due to the savings from unemployment benefits and earnings from income taxes. A gross approach to costing was used by first measuring and valuing costs at the level of the school and then disaggregating costs to the level of the child. The costs per child were calculated by dividing the total costs by the average number of children per school, because the interventions were provided at the school-level, and children were equally exposed to the interventions. All costs are expressed in 2016 euros. Costs were also converted to American dollars using the purchase power parity (PPP 2016, US$ 1 = € 0.82) (https://data.oecd.org/conversion/purchasing-power-parities-ppp.htm). Given the 1-year time horizon, discounting was not applied.

#### Scenario Analyses

While the initial delivery of school-based lifestyle interventions takes place during a research period, interventions are only embedded after research has ended. Until interventions become embedded into the school setting, learning-curve effects and efficiency improvements may occur, because more experience is obtained with the delivery of interventions (e.g., some activities may take less time). If interventions are sustained, they become to rely on more structural sources of funding. For this reason, it is interesting to examine whether organizational improvements are feasible (without compromising the intervention’s relative effectiveness) in order to increase funding opportunities. In the first scenario analysis, the costs of HPSF and PAS were explored for a hypothetical steady state. The steady state reflected a hypothetical situation in which the HPSF and PAS interventions are delivered at their full capacity, and learning curves and efficiency improvements were no longer applicable. Because a steady state was not yet reached, stakeholders were consulted to define the expected learning curves and efficiency improvements.

Potential areas for cost reduction were examined with a pedagogical staff scenario and an efficiency scenario. In the pedagogical staff scenario, it was assumed that lunch breaks and activities were guided by teaching assistants instead of pedagogical staff from external childcare partners. The efficiency scenario was constructed based on the views of stakeholders about the potential areas for further cost reductions. Stakeholders reported that potential cost reductions could be achieved by decreasing the time investments of external parties providing workshops (not considered as an essential intervention component) and volunteers (no volunteers needed in upper grades), and by reducing the costs of transport (cost reduction of 50% in the first year after implementation given the limited use of available budgets during the first year).

Furthermore, scenario analyses may be performed when parameters are uncertain. In the extended school day scenario, we relaxed the assumption that the additional school hours resulted in a cost offset due to the time being freed up for primary caregivers. In the extended school day scenario, we did not apply a cost offset as it was assumed that the extended school day also caused utility losses as primary caregivers might derive utility or wellbeing from being able to provide after-school care.

#### Sensitivity Analyses

Sensitivity analyses were conducted for the first year after implementation and for the hypothetical steady state. The main input parameters were varied to examine whether the costs per child were influenced by changes in resource consumption and unit prices. Input parameters were varied with ± 50% because volumes and unit prices were not empirically measured, and the uncertainty around the parameters was therefore unknown.

## Results

In this study, we developed a template to support cost calculations for lifestyle interventions in the primary school setting and illustrated its applicability.

### Template for Cost Calculation

Personnel costs contained the time investments of stakeholders that were compensated (e.g., salary, financial contribution) or not compensated. School personnel may invest time in the local planning, coordination, training (and substituting teachers during the training of personnel), delivery, and evaluation of interventions. The time investments of volunteers and primary caregivers belonged to the household sector. Negative costs may come into play when the school day is extended or when interventions are offered during the after-school hours, which results in time being freed up for the primary caregiver of the child (reduction in caregiver time). Personnel costs may apply to multiple stakeholder perspectives. This occurs when stakeholders outside the education sector receive subsidies or financial contributions to compensate their time investments (e.g., time investments of volunteers are costs to the household sector, and the financial compensation is a cost to the education sector). To avoid double counting when calculating the costs over multiple perspectives, it is important to only count the net costs for each stakeholder, being the value of time investments minus the received financial contributions. Overheads were not included in the framework as the proportion of overheads may vary between settings and stakeholder perspectives. However, it should be noted that taking account of overheads is highly relevant for the costing of interventions. An overview of the cost items of primary school-based lifestyle interventions is presented in Table 1.

**Table 1:** Cost items of primary school-based lifestyle interventions

Cost type	Cost item	Stakeholder perspective			
		Education	Household and leisure	Labor and social security	Healthcare
Personnel^C^	School personnel				
	Program coordinator	X			
	School project leader	X			
	Teacher	X			
	Teaching assistant	X			
	Volunteers, carers, and beneficiaries from the household sector	X	X^A^	X^A^	
	External parties from the leisure sector	X	X^A^		
	Personnel from the local government (e.g., cross-discipline coordinators)	X		X^A^	
	Personnel from the labor sector (e.g., pedagogical staff from childcare partners, catering personnel)	X		X^A^	
	Health staff (e.g., local health department)	X			X^A^
Materials	Transport	X			
	Accommodations	X			
	Food	X	X^B^		
	Curriculum materials	X			
	Monitoring equipment	X			
	Advertising and promotion	X			
	Accreditation and certification	X			
	Training materials for personnel	X			
	Communication and administration	X			

^A^There is a potential overlap in costs (see main text). To avoid double counting with the costs in the education sector, it is important to only count the net costs for each stakeholder, being the value of time investments minus the received financial contributions

^B^Food costs are a positive cost to the education sector and a negative cost to the household sector and do not indicate an overlap in costs

^C^Stakeholders may invest time for the local planning, coordination, training (and substituting teachers during the training of personnel), delivery, and recurrent evaluation. Furthermore, a per child fee may be required to compensate for the time investments of stakeholders. When beneficiaries of unemployment benefits are employed, this may lead to offsets in the household sector and in the social security sector (see main text)

### Cost Analysis of HPSF and PAS

From a societal perspective, the per child costs amounted to €2.7/day for HPSF and to €− 0.3/day for PAS during the first year after implementation (Table 2, results expressed in American dollars are available online). Because the fee for supervision during the lunch break (€1/child/day) was not charged in the first year after implementation, an additional cost reduction or financial contribution of €1.7/day (first year) is required to make the HPSF intervention cost-saving from a societal perspective. The highest costs were incurred by the education perspective (€8.7 (HPSF) and €4.0 (PAS) per day), whereas most of the cost reductions were received by the household and leisure perspective (€− 6.0 (HPSF) and €− 4.4 (PAS)). Given the education perspective, the personnel costs (HPSF, 69%; PAS, 94%) made up the largest proportion of the costs for HPSF and PAS.

**Table 2:** Per child costs of HPSF and PAS for the first year after implementation

Cost items for the first year after implementation	Time investment	Volume (per school)	Unit price	Stakeholder perspective									
				Education		Household and leisure		Labor and social security		Healthcare		Societal perspective	
				HPSF	PAS	HPSF	PAS	HPSF	PAS	HPSF	PAS	HPSF	PAS
Personnel													
Program coordinator	Coordination	4 schools: 1 FTE	€112,000/FTE^1^	€84	€84								
School project leaders	Coordination	HPSF: 0.5 FTE; PAS: 0.4 FTE	€65,000/FTE^1^	€97	€78								
Volunteers	Assisting during lunch break and activities		Financial compensation: HPSF: €12,645^1^ PAS: €8145^1^	€38	€24	€53^A^	€17^A^						
		Time investment HPSF: 12 volunteers, 1 h/day, 4 days/week (upper grades 5 days/week), 40 weeks PAS: 7 volunteers, 1 h/day, 3 days/week (upper grades 4 days/week), 40 weeks	Time investment €14/hour^4^										
Primary caregivers	Per child fee for supervision during lunch break	4 times/week, 40 weeks	€1/day not charged to carers^1^	€160	€160	€− 160	€− 160						
	Parental evaluation committee	5 times/year, 1 h, 10 persons	€14/h^4^			€2	€2						
	Value of the extended school hours	0.5 h freed up, 4 times/week 2 children / household	€14/hour^4^			€− 562	€− 562						
Beneficiaries of unemployment benefits	Preparing lunches as part of reintegration to the labor market	1 person, 15 h/week	NA^E^										
External parties from the leisure sector	Giving workshops		€6675^1^	€20	€20	€0^B^	€0^B^						
Cross-discipline coordinators from the local government	Organizing activities	1 FTE/4 schools	€50,000/FTE^1^	€37	€37			€0^C^	€0^C^				
Pedagogical staff from childcare partners	Guiding lunch break and activities	HPSF: 12 persons, 2 h/day, 4 days/week (upper grades 5 days/week), 40 weeks PAS: 8 persons, 1.5 h/day, 3 days/week (upper grades 4 days/week), 40 weeks	€65,000/FTE^1^	€524	€204			€0^D^	€0^D^				
Materials													
Transport			€7097^3^	€21	€21								
Accommodations		1 h, 4 days/week	€17.5/h^3^	€8	€8								
Food (including personnel from caterer)		HPSF: 4 times/week, 40 weeks	Cost: €2.46/child/day^3^ Offset: €− 1.86/day^5^	€394^F^		€− 298^F^							
Curriculum materials		1 set per year	€2500/school^1^	€7	€7								
Monitoring equipment		1 survey	€1200/survey^1^	€4	€4								
Total costs													
Net costs (per child/year)				€1394	€647	€− 965	€− 704	€0	€0	€0	€0	€429	€− 57
Personnel				€959	€606	€− 667	€− 704	€0	€0	€0	€0	€292	€− 96
Materials				€434	€41	€− 298	€0	€0	€0	€0	€0	€137	€41
Net costs (per child/day)				€8.7	€4.0	€− 6.0	€− 4.4	€0	€0	€0	€0	€2.7	€− 0.3

*HPSF* Healthy Primary School of the Future, *PAS* Physical Activity School; *FTE* full-time equivalent

Discrepancies between the sum of cost items may be due to rounding

^1^Budget “the Healthy Primary School of the Future”

^2^Productivity costs of paid labor (Zorginstituut Nederland 2015)

^3^Accounting data “the Healthy Primary School of the Future”

^4^Productivity costs of unpaid labor (Zorginstituut Nederland 2015)

^5^Household expenses on children’s lunches (NIBUD 2017)

^6^Minimum wage (“Minimumloon 2016”)

^7^Unemployment benefits (Rijksoverheid 2016)

^8^Income tax (Belastingdienst 2016)

^A^Value of time investment minus financial compensation

^B–D^Financial contributions fully compensated the time investments

^E^Assumed that offsets due to the reintegration of beneficiaries only applied to the steady state

^F^Food costs are a positive cost to the education sector and a negative cost to the household sector

### Scenario Analysis

With the steady-state assumptions such as the reductions in time investments of coordinators and the unit costs of lunches (Table 3, results expressed in American dollars are available online), the per child costs amounted to €1.0/day for HPSF and to €− 1.3/day for PAS. The costs for the education sector (€6.1 (HPSF) and €2.1 (PAS)) were fully compensated for PAS by the savings in the household and leisure sector (€− 5.0 (HPSF) and €− 3.4 (PAS)). In the hypothetical steady state, the HPSF intervention could become cost-saving from a societal perspective when the unit costs of lunches do not exceed the cost threshold of €1.0 per child per day, or when the costs of pedagogical staff will decrease by 70%.

**Table 3:** Per child costs of HPSF and PAS for a hypothetical steady state

Cost items for the steady state	Activities	Volume (per school)	Unit price	Stakeholder perspective									
				Education		Household and leisure		Labor and social security		Healthcare		Societal perspective	
				HPSF	PAS	HPSF	PAS	HPSF	PAS	HPSF	PAS	HPSF	PAS
Personnel													
Program coordinator	Coordination	4 schools: 0.25 FTE^A^	€112,000/FTE ^1^	€21	€21								
School project leaders	Coordination	0.25 FTE^A^	€65,000/FTE ^1^	€49	€49								
Volunteers	Assisting during lunch break and activities		Financial compensation:	€38	€24	€53^B^	€17^B^						
			HPSF: €12,645^1^ PAS: €8145^1^										
		Time investment HPSF: 12 volunteers, 1 h/day, 4 days/week (upper grades 5 days/week), 40 weeks PAS: 7 volunteers, 1 h/day, 3 days/week (upper grades 4 days/week), 40 weeks	Time investment €14/hour^4^										
Primary caregivers	Parental evaluation committee	5 times/year, 1 h, 10 persons	€14/h^4^			€2	€2						
	Value of the extended school hours	0.5 h freed up, 4 times/week 2 children/household	€14/hour^4^			€− 562	€− 562						
Beneficiaries of unemployment benefits	Preparing lunches as part of reintegration to the labor market	1 person, 15 h/week	Income level: €1525/month^6^ Unemployment benefits: €1320/month^7^; €8.80/hour^6^ Income taxes: 36.55%^8^			€− 3^C^	€0	€− 22^C^	€0				
External parties from the leisure sector	Giving workshops		€6675^1^	€20	€20	€0^D^	€0^D^						
Pedagogical staff from childcare partners	Guiding lunch break and activities	HPSF: 12 persons, 2 h/day, 4 days/week (upper grades 5 days/week), 40 weeks PAS: 8 persons, 1.5 h/day, 3 days/week (upper grades 4 days/week), 40 weeks	€65,000/FTE^1^	€524	€204			€0^E^	€0^E^				
Materials													
Food (including personnel from caterer)		HPSF: 4 times/week, 40 weeks	Cost: €2/child/day^A^ Offset: €1.86/day^5^	€320^F^	€0	€− 298^F^	€0						
Curriculum materials		1 set per year	€2500/school^1^	€7	€7								
Monitoring equipment		1 survey	€1200/survey^1^	€4	€4								
Total costs													
Net costs (per child/year)				€982	€328	€− 807	€− 544	€− 22	€0	€0	€0	€153	€215
Personnel				€651	€317	€− 510	€− 544	€− 22	€0	€0	€0	€120	€− 226
Materials				€331	€11	€− 298	€0	€0	€0	€0	€0	€33	€11
Net costs (per child/day)				€6.1	€2.1	€− 5.0	€− 3.4	€− 0.1	€0	€0	€0	€1.0	€− 1.3

*HPSF* Healthy Primary School of the Future; *PAS* Physical Activity School; *FTE* full-time equivalent.

Discrepancies between the sum of cost items may be due to rounding.

^1^Budget “the Healthy Primary School of the Future”

^2^Productivity costs of paid labor (Zorginstituut Nederland 2015)

^3^Accounting data “the Healthy Primary School of the Future”

^4^Productivity costs of unpaid labor (Zorginstituut Nederland 2015)

^5^Household expenses on children’s lunches (NIBUD 2017)

^6^Minimum wage (“Minimumloon 2016”)

^7^Unemployment benefits (Rijksoverheid 2016)

^8^Income tax (Belastingdienst 2016)

^A^Steady state assumption

^B^Value of time investment minus financial compensation

^C^Household: income level minus unemployment benefits; social security: savings from unemployment benefits and earnings from income taxes

^D–E^Financial contributions fully compensated the time investments

^F^Food costs are a positive cost to the education sector and a negative cost to the household sector

The pedagogical staff scenario led to the highest cost reductions (to €− 0.6 (HPSF) and €− 1.6 (PAS) in the first year after implementation) (Table 4, results expressed in American dollars are available online). Further research should be performed to assess whether these scenarios result cost reductions without compromising the intervention’s relative effectiveness.

**Table 4:** Scenario analysis (per child societal costs)

Scenario	Description	First year after implementation	Hypothetical steady state
Base-case		HPSF: €429 (€2.7/day)	
		PAS: €− 55 (€− 0.3/day)	
1.1. Hypothetical steady state	A situation that occurs on the long run, when interventions are delivered at their full capacity and learning curves, and efficiency improvements do not longer occur. Assumptions were defined by stakeholders.		HPSF: €153 (€1.0/day)
			PAS: €− 214 (€− ;1.3/day)
2.2. Pedagogical staff scenario	A scenario analysis to examine areas for further cost reductions. Lunch breaks and activities were guided by teaching assistants instead of pedagogical staff from external childcare partners.	HPSF: €− ;95 (€− ;0.6/day)	HPSF: €− ;371(€− ;2.3/day)
		PAS: €− ;259 (€− ;1.6/day)	AS: €− ;417 (€− ;2.6/day)
2.3. Efficiency scenario	Views of stakeholders about the potential areas for further cost reductions	HPSF: €353 (€2.2/day)	HPSF: €88 (€0.6/day)
		PAS: €− ;107 (€− ;0.7/day)	PAS: €− ;254 (€− ;1.6/day)
2.4. Extended school day scenario	A scenario analysis to examine the uncertainty about the cost item. It was assumed that the time offset for the primary caregiver due to the extended school day (saving) was fully compensated by the utility loss from being able to provide after-school care.	HPSF: €991 (€6.2/day)	HPSF: €715 (€4.5/day)
		PAS: €507 (€3.2/day)	PAS: €348 (€2.2/day)

*HPSF* Healthy Primary School of the Future; *PAS* Physical Activity School

The extended school day scenario drives up the societal per child costs from €2.7 per day to €6.2 (HPSF) and from €− 0.3 per day to €3.2 (PAS) in the first year after implementation.

### Sensitivity Analysis

**Fig. 1:**
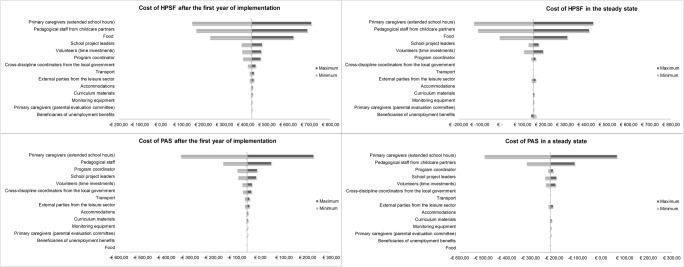
Tornado diagram (€/child/year). HPSF Healthy Primary School of the Future, PAS Physical Activity School

**Fig. 2:**
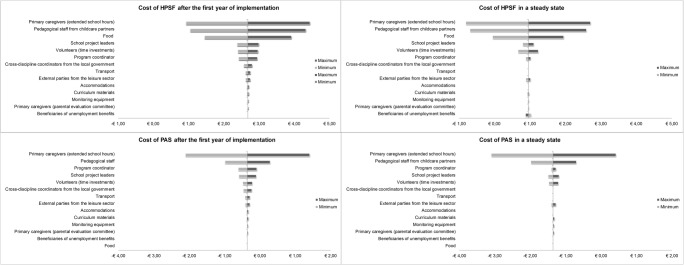
Tornado diagram (€/child/day). HPSF Healthy Primary School of the Future, PAS Physical Activity School

Figures 1 and 2 show how the costs per child vary with the upper and lower bounds on the key inputs (full tables available online). The time investments on after-school care in the household, the costs of pedagogical staff, and the unit costs of food were the main factors of influence.

## Discussion

The objective of this study was to develop a template for the costing of primary school-based lifestyle interventions in order to improve the quality and comparability of cost calculations. The framework listed the cost items of primary school-based lifestyle interventions and distinguished between the education, household and leisure, labor and social security, and health perspective. The framework proved useful for calculating the costs of HPSF and PAS: It helped to systematically identify the cost items, distinguish between stakeholder perspectives, and avoid the double counting of cost items. The societal costs were estimated at €2.7 and €− 0.3 per day in the first year after implementation and at €1.0 (HPSF) and €− 1.3 (PAS) for a hypothetical steady state, respectively. The education sector incurred the highest per child costs (€8.7 and €6.1/day (HPSF); €4.0 and €2.1/day (PAS)), which were fully compensated by the savings in the household sector for PAS (€− 4.4/day and €− 3.4/day), and almost fully compensated for HPSF (€− 6.0/day and €− 5.0/day).

Cost estimates are dependent on the methodological approaches and assumptions that are being used for making cost calculations. For example, we included delivery-related offsets as they may affect the opportunity costs to stakeholders. The impact of the extended school hours was included, assuming that the time freed up for the primary caregiver led to productivity gains. Previous cost-effectiveness studies on after-school interventions also accounted for the cost offsets on after-school care (Cradock et al. 2017; Wang, Sekine, Chen, Yamagami, and Kagamimori 2008). However, this approach can be debated as it only incorporates the costs of after-school care. Providing after-school care may also lead to gains in utility or wellbeing in primary caregivers (van den Berg, Brouwer, and Koopmanschap 2004). Combining these utility losses with the productivity gains would result in a lower cost offset. In addition, time offsets may not fully be translated in productivity gains. The scenario analysis showed that the offsets of the extended school day were of major influence on the intervention costs. Future research on the value of after-school care is warranted to be able to generate cost estimates that reflect true social opportunity costs.

Assumptions can also apply to the context of the cost analysis. Cost calculations are often made for interventions in a steady state, meaning that the intervention is implemented at its full effectiveness potential. These cost estimates can be used in economic evaluations to examine the full cost-effectiveness potential of interventions. On the other hand, estimating the intervention costs with the associated learning curves may be more relevant to inform implementation processes. Given the time and resource consuming processes for assessing learning curves, several studies assumed that interventions operated at steady-state conditions by excluding the one-off costs for start-up (Cobiac, Vos, and Veerman 2010; Moodie et al. 2010; Moodie et al. 2011). The resulting estimates do not take account of the potential learning effects and cost efficiencies (e.g., changes in time investments, improved capacity utilization, reduction in unit costs) that may occur over time (Anderson et al. 2007). In this study, stakeholders were consulted to define the expected learning curves and efficiency improvements. With both approaches (e.g., only excluding one-off costs versus defining a hypothetical steady state), the interventions costs may be underestimated or overestimated. The intervention costs should be monitored to examine whether the approaches can be used to produce accurate cost estimates.

The costs of HPSF and PAS were estimated for various perspectives in order to examine the cost distribution among stakeholders groups. The results showed large unbalances between the costs for education sector and the household sector (e.g., education perspective, €8.7/day; household perspective, €− 6.0/day (HPSF, first year)). In the Netherlands, the costs of the usual education program amounts to €32, per child per day (updated to 2016 prices) (Ministerie voor Onderwijs Cultuur en Wetenschap (OCW) 2014). The delivery of HPSF (incremental cost, €8.7/child/day) and PAS (incremental cost, €4.0/child/day) is associated with a 27 and 13% increase in the daily educational expenses, respectively. These results indicate that cost redistributions among stakeholder groups might be required to facilitate and support the implementation of the interventions. For example, it can be argued that a per child fee may be charged to primary caregivers who can afford it, as the societal costs of €2.7 (HPSF first year after implementation) are lower than the total of the general per child fees for supervision during the lunch break (€1/child/day) and the household expenses that are usually required for lunches and morning snacks for children in the regular school curriculum (€1.8/child/day) (NIBUD 2017).

In addition to the offsets of the extended school day, the sensitivity analysis showed that personnel costs had a major influence on the costs of the HPSF and PAS interventions. In several countries, primary education, childcare, and after-school care are becoming more integrated with each other (Oostdam, Tavecchio, Nøhr, and Ex 2014). A close collaboration between multiple professionals and a redistribution of their tasks (e.g., supervision during lunch break, assisting in the classroom, providing after-school care) may possibly lead the way to obtain further cost reductions and facilitate the implementation of lifestyle interventions in primary schools.

The results showed that the delivery of HPSF is a more costly alternative compared to PAS. In addition to the costing study, the relative effectiveness of both interventions on outcomes including health, wellbeing, and educational outcomes will be evaluated to examine whether the additional costs of HPSF and PAS represent value for money when compared to the regular school curriculum.

## Conclusions

The template proved helpful in calculating the costs of school-based lifestyle interventions and distinguishing between various stakeholder perspectives. The results showed that the delivery of HPSF is a more costly alternative compared to PAS. The education sector incurred the highest per child costs, which were fully compensated by the savings in the household sector for PAS, and almost fully compensated for HPSF. Whether the additional costs of HPSF over PAS represent value for money depends on the relative effectiveness of the interventions.

## Electronic supplementary material

ESM 1 (DOCX 29 kb)

ESM 2 (DOCX 30 kb)

ESM 3 (DOCX 29 kb)

ESM 4 (DOCX 17 kb)
