# Supplementary material for: A Cost Analysis of School-Based Lifestyle Interventions
Source: Prev Sci. 2018 May 31;19(6):716–27. doi: 10.1007/s11121-018-0918-1 (PMC6599187; doi:10.1007/s11121-018-0918-1)
Supplement: Supplementary file 1 — (DOCX 29 kb) [file 11121_2018_918_MOESM1_ESM.docx]

Supplemental materials

Table S1. Sensitivity analysis (€/child/year)

| Input (per school) | | Cost of the item (HPSF) | Societal costs HPSF | Difference with base case | Cost of the item (PAS) | Societal costs PAS | | Difference with base case |
| --- | --- | --- | --- | --- | --- | --- | --- | --- |
| Program coordinator | | | | | | | | |
| *First year after implementation* | Base case (0.25 FTE) | €84 | €429 | NA | €84 | €-55 | | NA |
|  | -50% (0.13 FTE) | €42 | €387 | -9.7% | €42 | €-97 | | -75.9% |
|  | +50% (0.38 FTE) | €125 | €471 | 9.7% | €125 | €-13 | | 75.9% |
| *Hypothetical steady state* | Base case (0.06 FTE) | €21 | €153 | NA | €21 | €-214 | | NA |
|  | -50% (0.03 FTE) | €10 | €143 | -6.8% | €10 | €-224 | | -4.9% |
|  | +50% (0.09 FTE) | €31 | €164 | 6.8% | €31 | €-203 | | 4.9% |
| School project leaders | | | | | | | | |
| *First year after implementation* | Base case (0.5; 0.4 FTE) | €97 | €429 | NA | €78 | €-55 | NA | |
|  | -50% (0.25; 0.20 FTE) | €49 | €380 | -11.3% | €39 | €-94 | -70.5% | |
|  | +50% (0.75 ; 0.60 FTE) | €146 | €478 | 11.3% | €116 | €-16 | 70.5% | |
| *Hypothetical steady state* | Base case (0.25FTE) | €48 | €153 | NA | €49 | €-214 | NA | |
|  | -50% (0.13 FTE) | €24 | €129 | -15.8% | €24 | €-238 | -11.4% | |
|  | +50% (0.38 FTE) | €73 | €177 | 15.8% | €73 | €-189 | 11.4% | |
| Volunteers | | | | | | | | |
| *First year after implementation* | Base case (see Table 2) | See Table 2 | €429 | NA | See Table 2 | €-55 | NA | |
|  | -50% |  | €384 | -10.5% |  | €-76 | -38.0% | |
|  | +50% |  | €474 | 10.5% |  | €-34 | 38.0% | |
| *Hypothetical steady state* | Base case (see Table 3) | See Table 3 | €153 | NA | See Table 3 | €-214 | NA | |
|  | -50% |  | €108 | -29.5% |  | €-235 | -9.8% | |
|  | +50% |  | €198 | 29.5% |  | €-193 | 9.8% | |
| Primary caregiver (parental evaluation committee | | | | | | | | |
| *First year after implementation* | Base case (10 carers) | €2 | €429 | NA | €2 | €-55 | NA | |
|  | -50% (5 carers) | €1 | €428 | -0.2% | €1 | €-56 | -1.9% | |
|  | +50% (15 carers) | €3 | €430 | 0.2% | €3 | €-54 | 1.9% | |
| *Hypothetical steady state* | Base case (10 carers) | €2 | €153 | NA | €2 | €-214 | NA | |
|  | -50% (5 carers) | €1 | €152 | -0.7% | €1 | €-215 | -0.5% | |
|  | +50% (15 carers) | €3 | €154 | 0.7% | €3 | €-213 | 0.5% | |

| Input (per school) | | Cost of the item (HPSF) | Societal costs HPSF | Difference with base case | | Cost of the item (PAS) | | Societal costs PAS | | | Difference with base case | |
| --- | --- | --- | --- | --- | --- | --- | --- | --- | --- | --- | --- | --- |
| Primary caregiver (extended school hours) | | | | | | | | | | | | |
| *First year after implementation* | Base case (80 hours/carer/year) | €-562 | €429 | NA | | €-562 | | €-55 | | | NA | |
|  | -50% (40 hours/carer/year) | €-281 | €710 | 65.5% | | €-281 | | €226 | | | 510.3% | |
|  | +50% (120 hours/carer/year) | €-843 | €148 | -65.5% | | €-843 | | €-336 | | | -510.3% | |
| *Hypothetical steady state* | Base case (80 hours/carer/year) | €-562 | €153 | NA | | €-562 | | €-214 | | | NA | |
|  | -50% (40 hours/carer/year) | €-281 | €434 | -183.5% | | €-281 | | €67 | | | 131.4% | |
|  | +50% (120 hours/carer/year) | €-843 | €-128 | 183.5% | | €-843 | | €-495 | | | -131.4% | |
| Beneficiaries of unemployment benefits | | | | | | | | | | | | |
| *Hypothetical steady state* | Base case (15 personhours/week) | household:€-2,75  social sec: €-21,25 | €153 | NA | | NA | | NA | | | | NA |
|  | -50% (7,5 personhours/week) | household:€-1,37  social sec: €-10,76 | €165 | 7.9% | | NA | | NA | | | | NA |
|  | +50% (22,5 personhours/week) | household:€-4,12  social sec: €-32,28 | €141 | -7.9% | | NA | | NA | | | | NA |
| External parties from the leisure sector | |  |  |  | |  | |  | | | |  |
| *First year after implementation* | Base case (€3.086) | €20 | €429 | NA | | €9 | | €-55 | | | | NA |
|  | -50% (€1.543) | €10 | €419 | -2.3% | | €5 | | €-65 | | | | -18.1% |
|  | +50% (€4.629) | €30 | €439 | 2.3% | | €14 | | €-45 | | | | 18.1% |
| *Hypothetical steady state* | Base case (€6.675) | €20 | €153 | NA | | €20 | | €-214 | | | | NA |
|  | -50% (€3.338) | €10 | €143 | -6.5% | | €10 | | €-224 | | | | -4.7% |
|  | +50% (€10.013) | €30 | €163 | 6.5% | | €30 | | €-204 | | | | 4.7% |
| Cross-discipline coordinators from the local government | |  | | |  | |  | |  |  | | |
| *First year after implementation* | Base case (0.25 FTE) | €37 | €429 | NA | | €37 | | €-55 | | | | NA |
|  | -50% (0.13 FTE) | €19 | €410 | -4.3% | | €19 | | €-74 | | | | -33.9% |
|  | +50% (0.38 FTE) | €56 | €448 | 4.3% | | €56 | | €-37 | | | | 33.9% |
| Pedagogical staff from childcare partners | | | | | | | | | | | | |
| *First year after implementation* | Base case (see Table 2) | €524 | €429 | NA | | €204 | | €-55 | | | | NA |
|  | -50% | €262 | €167 | -61.1% | | €102 | | €-157 | | | | -185.1% |
|  | +50% | €786 | €691 | 61.1% | | €306 | | €47 | | | | 185.1% |
| *Hypothetical steady state* | Base case (see Table 3) | €524 | €153 | NA | | €204 | | €-214 | | | | NA |
|  | -50% | €262 | €-10 | -171.1% | | €102 | | €-316 | | | | -47.7% |
|  | +50% | €786 | €415 | 171.1% | | €306 | | €-112 | | | | 47.7% |

Table S1. [continued]

Table S1. [continued]

| Input (per school) | | Cost of the item (HPSF) | Societal costs HPSF | Difference with base case | Cost of the item (PAS) | Societal costs PAS | Difference with base case |
| --- | --- | --- | --- | --- | --- | --- | --- |
| Transport | | | | | | | |
| *First year after implementation* | Base case (€7.097) | €21 | €429 | NA | €21 | €-55 | NA |
|  | -50% (€3.549) | €11 | €418 | -2.5% | €11 | €-66 | -19.2% |
|  | +50% (€10.645) | €32 | €440 | 2.5% | €32 | €-44 | 19.2% |
| Accommodations | | | | | | | |
| *First year after implementation* | Base case (see Table 2) | €8 | €429 | NA | €8 | €-55 | NA |
|  | -50% | €4 | €425 | -1.0% | €4 | €-59 | -7.6% |
|  | +50% | €13 | €433 | 1.0% | €13 | €-51 | 7.6% |
| Food (education) | | | | | | | |
| *First year after implementation* | Base case (€2.46/child/day) | €394 | €429 | NA | NA | NA | NA |
|  | -50% (€2.13/child/day) | €197 | €232 | -45.9% | NA | NA | NA |
|  | +50% (€3.69/child/day) | €590 | €626 | 45.9% | NA | NA | NA |
| *Hypothetical steady state* | Base case (€2/child/day) | €320 | €153 | NA | NA | NA | NA |
|  | -50% (€1/child/day) | €160 | €-7 | -104.5% | NA | NA | NA |
|  | +50% (€3/child/day) | €480 | €313 | 104.5% | NA | NA | NA |
| Curriculum materials | | | | | | | |
| *First year after implementation* | Base case (€2.500/set) | €7 | €429 | NA | €7 | €-55 | NA |
|  | -50% (€1.250/set) | €4 | €426 | -0.9% | €4 | €-59 | -7% |
|  | +50% (€3.750/set) | €11 | €433 | 0.9% | €11 | €-51 | 7% |
| *Hypothetical steady state* | Base case (€2.500/set) | €7 | €153 | NA | €7 | €-214 | NA |
|  | -50% (€1.250/set) | €4 | €149 | -2.4% | €4 | €-217 | -1.7% |
|  | +50% (€3.750/set) | €11 | €157 | 2.4% | €11 | €210 | 1.7% |
| Monitoring equipment | | | | | | | |
| *First year after implementation* | Base case (€1.200/survey) | €4 | €429 | NA | €4 | €-55 | NA |
|  | -50% (€600/survey) | €2 | €427 | -0.4% | €2 | €-57 | -3.3% |
|  | +50% (€1.800/survey) | €5 | €431 | 0.4% | €5 | €-53 | 3.3% |
| *Hypothetical steady state* | Base case (€1.200/survey) | €4 | €153 | NA | €4 | €-214 | NA |
|  | -50% (€600/survey) | €2 | €151 | -1.2% | €2 | €-215 | -0.8% |
|  | +50% (€1.800/survey) | €5 | €155 | 1.2% | €5 | €-212 | 0.8% |

FTE = full-time equivalent; HPSF = Healthy Primary School of the Future; PAS = Physical Activity School
