# Supplementary material for: A Cost Analysis of School-Based Lifestyle Interventions
Source: Prev Sci. 2018 May 31;19(6):716–27. doi: 10.1007/s11121-018-0918-1 (PMC6599187; doi:10.1007/s11121-018-0918-1)
Supplement: Supplementary file 2 — (DOCX 30 kb) [file 11121_2018_918_MOESM2_ESM.docx]

**Table S2.** Per child costs of HPSF and PAS for the first year after implementation expressed in American dollars

| **Cost items for the first year after implementation** | | **Time investment** | | **Volume** (per school) | | | **Unit price** | **Stakeholder perspective** | | | | | | | | | |
| --- | --- | --- | --- | --- | --- | --- | --- | --- | --- | --- | --- | --- | --- | --- | --- | --- | --- |
|  |  |  |  |  |  |  |  | **Education** | | **Household & leisure** | | **Labour & social security** | | **Healthcare** | | **Societal perspective** | |
|  |  |  |  |  |  |  |  | **HPSF** | **PAS** | **HPSF** | **PAS** | **HPSF** | **PAS** | **HPSF** | **PAS** | **HPSF** | **PAS** |
| ***Personnel*** | |  | |  | | |  |  |  |  | |  |  |  |  |  |  |
| Program coordinator | | Coordination | | 4 schools: 1 FTE | | | $137,000 / FTE ^1^ | $102 | $102 |  |  |  |  |  |  |  |  |
| School project leaders | | Coordination | | HPSF: 0.5 FTE ; PAS: 0.4 FTE | | | $79,000 / FTE ^1^ | $118 | $95 |  |  |  |  |  |  |  |  |
| Volunteers | | Assisting during lunch break and activities | |  | | | Financial compensation:  HPSF: $15,420 ^1^  PAS: $9,932 ^1^ | $46 | $29 | $65 ^A^ | $21 ^A^ |  |  |  |  |  |  |
|  |  |  |  | Time investment  HPSF: 12 volunteers, 1 hour/day,  4 days/week (upper grades 5 days/week), 40 weeks PAS: 7 volunteers, 1 hour/day,  3 days/week (upper grades 4 days/week), 40 weeks | | | Time investment  $17 / hour ^4^ |  |  |  |  |  |  |  |  |  |  |
| Primary caregivers | | Per child fee for supervision during lunch break | | 4 times / week, 40 weeks | | | $1 / day not charged to carers ^1^ | $195 | $195 | $-195 | $-195 |  |  |  |  |  |  |
|  |  | Parental evaluation committee | | 5 times / year, 1 hours, 10 persons | | | $17/ hour ^4^ |  |  | $2 | $2 |  |  |  |  |  |  |
|  |  | Value of the extended school hours | | 0.5 hour freed-up, 4 times / week 2 children / household | | | $17 / hour ^4^ |  |  | $-685 | $-685 |  |  |  |  |  |  |
| Beneficiaries of unemployment benefits | | Preparing lunches as part of reintegration to the labour market | | 1 person, 15 hours/week | | | NA ^E^ |  |  |  |  |  |  |  |  |  |  |
| External parties from the leisure sector | | Giving workshops | |  | | | $8,140 ^1^ | $24 | $24 | $0 ^B^ | $0 ^B^ |  |  |  |  |  |  |
| Cross-discipline coordinators from the local government | | Organizing activities | | 1 FTE / 4 schools | | | $60,975 / FTE ^1^ | $45 | $45 |  |  | $0 ^C^ | $0 ^C^ |  |  |  |  |
| Pedagogical staff from childcare partners | | Guiding lunch break and activities | | HPSF: 12 persons, 2 hours/day,  4 days/week (upper grades 5 days/week), 40 weeks PAS: 8 persons, 1.5 hours/day,  3 days/week (upper grades 4 days/week), 40 weeks | | | $79,268/ FTE ^1^ | $639 | $249 |  |  | $0 ^D^ | $0 ^D^ |  |  |  |  |
| ***Materials*** | |  | |  | | |  |  |  |  |  |  |  |  |  |  |  |
| Transport | |  | |  | | | $8,655 ^3^ | $26 | $26 |  |  |  |  |  |  |  |  |
| Accommodations | |  | | 1 hour, 4 days/week | | | $21/hour ^3^ | $10 | $10 |  |  |  |  |  |  |  |  |
| Food (including personnel from caterer) | | | | HPSF: 4 times / week, 40 weeks | | | Cost: $3 / child / day ^3^  Offset: $-2.27 / day ^5^ | $480 ^F^ |  | $-363 ^F^ |  |  |  |  |  |  |  |
| Curriculum materials |  | | | 1 set per year | | | $3,049 / school ^1^ | $9 | $9 |  |  |  |  |  |  |  |  |
| Monitoring equipment |  | | | 1 survey | | | $1,463/ survey ^1^ | $5 | $5 |  |  |  |  |  |  |  |  |
| ***Total costs*** |  | | |  | | |  |  |  |  |  |  |  |  |  |  |  |
| **Net costs (per child/year)** | | | | | | | | $1700 | $789 | $-1177 | $-859 | $0 | $0 | $0 | $0 | **$523** | **$-70** |
| Personnel | | |  | |  |  | | $1170 | $739 | $-813 | $-859 | $0 | $0 | $0 | $0 | $356 | $-117 |
| Materials | | |  | |  |  | | $529 | $50 | $-363 | $0 | $0 | $0 | $0 | $0 | $167 | $50 |
| **Net costs (per child/day)** | | |  | |  |  | | $10.6 | $4.9 | $-7.3 | $-5.4 | $0 | $0 | $0 | $0 | $3.3 | $-0.4 |

HPSF = Healthy Primary School of the Future; PAS = Physical Activity School; FTE = full-time equivalent.
Discrepancies between the sum of cost items may be due to rounding.
^1^ Budget ‘the Healthy Primary School of the Future’.
^2^ Productivity costs of paid labour (Zorginstituut Nederland 2015).
^3^ Accounting data ‘the Healthy Primary School of the Future’.
^4^ Productivity costs of unpaid labour (Zorginstituut Nederland 2015).
^5^ Household expenses on children’s lunches (NIBUD 2017).
^6^  Minimum wage ("Minimumloon 2016").
^7^ Unemployment benefits (Rijksoverheid 2016).
^8^ Income tax (Belastingdienst 2016).
^A^ Value of time investment minus financial compensation.
^B-D^ Financial contributions fully compensated the time investments.
^E^ Assumed that offsets due to the reintegration of beneficiaries only applied to the steady state.
^F^ Food costs are a positive cost to the education sector and a negative cost to the household sector.
