# Supplementary material for: A Cost Analysis of School-Based Lifestyle Interventions
Source: Prev Sci. 2018 May 31;19(6):716–27. doi: 10.1007/s11121-018-0918-1 (PMC6599187; doi:10.1007/s11121-018-0918-1)
Supplement: Supplementary file 3 — (DOCX 29 kb) [file 11121_2018_918_MOESM3_ESM.docx]

| **Cost items for the steady state** | | **Activities** | | **Volume** (per school) | | **Unit price** | **Stakeholder perspective** | | | | | | | | | |
| --- | --- | --- | --- | --- | --- | --- | --- | --- | --- | --- | --- | --- | --- | --- | --- | --- |
|  | |  | |  | |  | **Education** | | **Household & leisure** | | **Labour & social security** | | **Healthcare** | | **Societal perspective** | |
|  | |  |  |  |  |  | **HPSF** | **PAS** | **HPSF** | **PAS** | **HPSF** | **PAS** | **HPSF** | **PAS** | **HPSF** | **PAS** |
| Personnel | |  | |  | |  |  |  |  |  |  |  |  |  |  |  |
| Program coordinator | | Coordination | | 4 schools: 0.25 FTE ^A^ | | $137,000 / FTE ^1^ | $26 | $26 |  |  |  |  |  |  |  |  |
| School project leaders | | Coordination | | 0.25 FTE ^A^ | | $79,000 / FTE ^1^ | $60 | $60 |  |  |  |  |  |  |  |  |
| Volunteers | | Assisting during lunch break and activities | |  | | Financial compensation:  HPSF: $15,420 ^1^  PAS: $9,932 ^1^ | $46 | $29 | $65 ^B^ | $21 ^B^ |  |  |  |  |  |  |
|  |  |  |  | Time investment  HPSF: 12 volunteers, 1 hour/day,  4 days/week (upper grades 5 days/week), 40 weeks PAS: 7 volunteers, 1 hour/day,  3 days/week (upper grades 4 days/week), 40 weeks | | Time investment  $17 / hour ^4^ |  |  |  |  |  |  |  |  |  |  |
| Primary caregivers | | Parental evaluation committee | | 5 times / year, 1 hours, 10 persons | | $17 / hour ^4^ |  |  | $2 | $2 |  |  |  |  |  |  |
|  |  | Value of the extended school hours | | 0.5 hour freed-up, 4 times / week  2 children / household | | $17 / hour ^4^ |  |  | $-685 | $-685 |  |  |  |  |  |  |
| Beneficiaries of unemployment benefits | | Preparing lunches as part of reintegration to the labour market | | 1 person, 15 hours/week | | Income level: $1,860 / month ^6,^ Unemployment benefits: $1,610 / month^7^ ; $10.73 / hour ^6^  Income taxes: 36.55% ^8^ |  |  | $-4 ^C^ | €0 | $-27 ^C^ | €0 |  |  |  |  |
| External parties from the leisure sector | | Giving workshops | |  | | $8,140 ^1^ | $24 | $24 | $0 ^D^ | $0 ^D^ |  |  |  |  |  |  |
| Pedagogical staff from childcare partners | | Guiding lunch break and activities | | HPSF: 12 persons, 2 hours/day,  4 days/week (upper grades 5 days/week), 40 weeks PAS: 8 persons, 1.5 hour/day,  3 days/week (upper grades 4 days/week), 40 weeks | | $79,268/ FTE ^1^ | $639 | $249 |  |  | $0 ^E^ | $0 ^E^ |  |  |  |  |
| Materials | | | |  | |  |  |  |  |  |  |  |  |  |  |  |
| Food (including personnel from caterer) | | | | HPSF: 4 times / week, 40 weeks | | Cost: $2.44 / child / day ^A^  Offset: $2.27 / day ^5^ | $390 ^F^ | €0 | $-485 ^F^ | €0 |  |  |  |  |  |  |
| Curriculum materials | | | | 1 set per year | | $3,049 / school ^1^ | $9 | $9 |  |  |  |  |  |  |  |  |
| Monitoring equipment | | | | 1 survey | | $1,463 / survey ^1^ | $5 | $5 |  |  |  |  |  |  |  |  |
| Total costs |  | | |  | |  |  |  |  |  |  |  |  |  |  |  |
| **Net costs (per child/year)** | | | | | | | $1,198 | $400 | $-984 | $-663 | $-27 | $0 | $0 | $0€0 | **$187** | **$262** |
| Personnel | | |  | |  | | $794 | $387 | $-622 | $-663 | $-27 | $0 | $0 | $0 | $146 | $-276 |
| Materials | | | | |  | | $404 | $13 | $-363 | $0 | $0 | $0 | $0 | $0 | $40 | $13 |
| **Net costs (per child/day)** | | |  | |  | | $7.4 | $2.6 | $-6.1 | $-4.1 | $-0.1 | $0 | $0 | $0 | $1.2 | $-1.6 |

**Table S3.** Per child costs of HPSF and PAS for a hypothetical steady state expressed in American dollars

HPSF = Healthy Primary School of the Future; PAS = Physical Activity School; FTE = full-time equivalent.

Discrepancies between the sum of cost items may be due to rounding.
^1^ Budget ‘the Healthy Primary School of the Future’.
^2^ Productivity costs of paid labour (Zorginstituut Nederland 2015).
^3^ Accounting data ‘the Healthy Primary School of the Future’.
^4^ Productivity costs of unpaid labour (Zorginstituut Nederland 2015).
^5^ Household expenses on children’s lunches (NIBUD 2017).
^6^  Minimum wage ("Minimumloon 2016").
^7^ Unemployment benefits (Rijksoverheid 2016).
^8^ Income tax (Belastingdienst 2016).
^A^ Steady state assumption. ^B^ Value of time investment minus financial compensation.
^C^ Household: income level minus unemployment benefits ; social security: savings from unemployment benefits and earnings from income taxes.
^D-E^ Financial contributions fully compensated the time investments.
^F^ Food costs are a positive cost to the education sector and a negative cost to the household sector.
