# Supplementary material for: A Cost Analysis of School-Based Lifestyle Interventions
Source: Prev Sci. 2018 May 31;19(6):716–27. doi: 10.1007/s11121-018-0918-1 (PMC6599187; doi:10.1007/s11121-018-0918-1)
Supplement: Supplementary file 4 — (DOCX 17 kb) [file 11121_2018_918_MOESM4_ESM.docx]

Table S4. Scenario analysis (per child societal costs) expressed in American dollars

| **Scenario** | **Description** | **First year after implementation** | **Hypothetical steady state** |
| --- | --- | --- | --- |
| Base-case |  | HPSF: $523 ($3.3/day) PAS: $-67 ($-0.4/day) |  |
| 1. Hypothetical steady state | A situation that occurs on the long run, when interventions are delivered at their full capacity and learning curves and efficiency improvements do not longer occur. Assumptions were defined by stakeholders. |  | HPSF: $187($1.2€1.0/day) PAS: $-261 ($-1.6/day) |
| 1. Pedagogical staff scenario | A scenario analysis to examine areas for further cost reductions. Lunch breaks and activities were guided by teaching assistants instead of pedagogical staff from external childcare partners. | HPSF: $-116 ($-0.7/day) PAS: $-316 ($-2.0/day) | HPSF: $-452($-2.8/day) PAS: $-509 ($-3.2/day) |
| 1. Efficiency scenario | Views of stakeholders about the potential areas for further cost reductions. | HPSF: $430 ($2.7/day) PAS: $-130 ($-0.9/day) | HPSF: $107 ($0.7/day) PAS: $-310 ($-2.0/day) |
| 1. Extended school day scenario | A scenario analysis to examine the uncertainty about the cost item. It was assumed that the time offset for the primary caregiver due to the extended school day (saving) was fully compensated by the utility loss from being able to provide after-school care. | HPSF: $1,209 ($7.6/day) PAS: $618 ($3.9/day) | HPSF: $872 ($5.5/day) PAS: $424 ($2.7/day) |

HPSF = Healthy Primary School of the Future; PAS = Physical Activity School
